# Motor Cortex Stimulation for Pain Relief: Do Corollary Discharges Play a Role?

**DOI:** 10.3389/fnhum.2016.00323

**Published:** 2016-06-28

**Authors:** Joaquim P. Brasil-Neto

**Affiliations:** Laboratory of Neurosciences and Behavior, Department of Physiological Sciences, Universidade de BrasíliaBrasília, Brazil

**Keywords:** transcranial direct current stimulation (tDCS), chronic pain, pain neuromatrix, transcranial magnetic stimulation (TMS), motor cortex stimulation

## Abstract

Both invasive and non-invasive motor cortex stimulation techniques have been successfully employed in the treatment of chronic pain, but the precise mechanism of action of such treatments is not fully understood. It has been hypothesized that a mismatch of normal interaction between motor intention and sensory feedback may result in central pain. Sensory feedback may come from peripheral nerves, vision and also from corollary discharges originating from the motor cortex itself. Therefore, a possible mechanism of action of motor cortex stimulation might be corollary discharge reinforcement, which could counterbalance sensory feedback deficiency. In other instances, primary deficiency in the production of corollary discharges by the motor cortex might be the culprit and stimulation of cortical motor areas might then be beneficial by enhancing production of such discharges. Here we review evidence for a possible role of motor cortex corollary discharges upon both the pathophysiology and the response to motor cortex stimulation of different types of chronic pain. We further suggest that the right dorsolateral prefrontal cortex (DLPC), thought to constantly monitor incongruity between corollary discharges, vision and proprioception, might be an interesting target for non-invasive neuromodulation in cases of chronic neuropathic pain.

## Introduction

Chronic pain usually presents a therapeutic challenge. Its pathophysiology, however, is often obscure. Recently, there have been important advances in the understanding of chronic pain, and central mechanisms have been increasingly implicated in its initiation and perpetuation (Melzack, 1990, 2001; Harris, 1999; McCabe et al., 2005, 2008).

The first reports of chronic pain control by means of motor cortex stimulation were published more than two decades ago (Tsubokawa et al., 1991a,b). Since then, favorable effects of motor cortex stimulation upon chronic pain have been repeatedly reported, both with invasive and non-invasive techniques (García-Larrea et al., 1999; Nguyen et al., 2000; Brown, 2001; Nuti et al., 2005; Cioni and Meglio, 2007; Klein et al., 2015). The neurosurgical implantation of epidural or subdural electrodes yields the best results, but non-invasive techniques such as transcranial magnetic stimulation (TMS) and transcranial direct current stimulation (tDCS) pose fewer risks to the patient and have been increasingly studied (Klein et al., 2015).

The mechanisms of action of motor cortex stimulation for pain relief are not well understood. However, it has been demonstrated that repeated cortical stimulation, by various techniques, is capable of inducing cortical excitability changes (Hoogendam et al., 2010). In addition to that, it has been shown that, even in the adult brain, changes in sensory afferences due to disease or experimental manipulation lead to cortical reorganization (Merzenich et al., 1983; Sanes et al., 1988; Donoghue et al., 1990).

Several central nervous system (CNS) structures constitute the pain neuromatrix (Melzack, 1990, 2001). According to this theory, chronic pain should not be conceived as a direct consequence of noxious stimulation acting upon sensory pathways, but rather as the result of complex processing of information in the neuromatrix, influenced by its existing synaptic architecture, which is determined by genetic and sensory factors, as well as by influences from other parts of the brain. Significant cortical reorganization might, therefore, strongly influence pain processing in the neuromatrix.

Here, we review clinical and experimental evidence of an important role for cortical reorganization in the pathophysiology of chronic pain. We propose that, in physiological induction of motor cortex plasticity, as in the case of motor learning, the mechanisms responsible for sensory-motor integration remain intact, whereas in disease conditions that same plasticity may be maladaptive and lead to conflict between motor intention and sensory feedback. It has already been suggested that such conflict might lead to chronic pain (Harris, 1999; McCabe et al., 2005; Ramachandran et al., 2007).

## Corollary Discharges and Central Pain

Corollary discharges play a role in attenuating perception of voluntarily generated movement and also of self-inflicted pain (Berner et al., 2007; Voss et al., 2007; Therrien et al., 2011; Wang et al., 2011). This is similar to the suppression of vision during voluntary saccadic eye movements to avoid blurred vision and to the attenuation of auditory perception during speech. Both phenomena are produced by corollary (“re-afferent”) discharges.

In an interesting experiment, McCabe et al. (2005) studied 41 healthy adult volunteers without a history of motor or proprioceptive disorders who performed a series of bilateral upper and lower limb movements while viewing a mirror or a whiteboard, which created varied degrees of sensory-motor conflict during congruent and incongruent limb movements. Sixty-six percent of their subjects reported anomalous sensations in the limbs during performance of the incongruent condition; when they reported pain, this was described as numbness, pins and needles, moderate aching and/or a definite pain.

Harris (1999) hypothesized that central pain might result from an incongruity between intention to move, visual feedback and proprioception. The same author also proposed the existence of a cortical incongruity monitoring region in the right cerebral hemisphere that would also be responsible for the production of nausea in cases of conflict between vestibular and visual afferences. An interesting hint pointing towards a common mechanism implicated in nausea and painful sensations arising from sensory conflict is the analgesic effect of motion-sickness drugs, such as scopolamine and orphenadrine, in some cases of chronic pain (Goldstein, 2002).

Intention to move, as described by Harris (1999), probably relates to corollary discharges in the motor system. We might then substitute corollary discharges for intention to move and hypothesize that central pain might arise whenever mismatches occur between motor corollary discharges, visual feedback and proprioception. According to the PET studies of relative regional cerebral blood flow performed by Fink et al. (1999), “a ventral right lateral prefrontal region is primarily activated by discrepancies between signals from sensory systems, while a more dorsal area in right lateral prefrontal cortex is activated when actions must be maintained in the face of a conflict between intention and sensory outcome”.

### Transcranial Magnetic Stimulation and Corollary Discharges

Patients sometimes describe the illusion of movement of paralyzed limbs during TMS, a fact that might be explained by production of corollary discharges.

Ellaway et al. (2004) compared the timing of perception of peripherally produced muscle twitches in response to nerve electrical stimulation to that of similar twitches evoked centrally by TMS. Since perception of TMS-evoked twitches occurred, on average, 20 ms later than was the case for those produced by direct nerve stimulation, the authors concluded that the sensation of movement elicited by TMS was due to proprioceptive feedback rather than to intracortical corollary discharges.

In a more recent study, however, Christensen et al. (2010) studied TMS-induced sensation of movement of completely anesthetized limbs. They used repetitive TMS at a frequency of 20 Hz. Afferent and efferent neural signaling was abolished in the arm with ischemic nerve block, and in the leg with spinal nerve block. Under those conditions, they were able to demonstrate persistent sensation of movement, thus confirming its central origin. Both dorsal premotor and motor cortical stimulation produced such corollary discharges, but dorsal premotor cortex stimulation was more effective than motor cortex stimulation. Their conclusion was that “repetitive TMS over dorsal premotor cortex produces a corollary discharge that is perceived as movement”.

## Examples of Chronic Central Pain and Possible Sensory-Motor Mismatches

### Phantom Pain

A remarkable example of dissociation of motor intention and sensory feedback is given by the phantom of an amputated limb. Such phantoms are frequently painful (Ramachandran et al., 2007). In such a situation, the patient vividly perceives the phantom limb and may or may not be able to move it. In both cases, there is a mismatch between intention to move and the non-existent sensory feedback. It has been pointed out that paralyzed phantoms are usually painful (Ramachandran et al., 2007). Remarkably, when mirror therapy was used by Ramachandran et al. (2007) to provide visual feedback of the moving phantom (albeit artificially), many patients experienced striking decrease or complete resolution of their phantom pain.

In such cases, there is maladaptive plasticity of the somatotopic representation of the missing limb in the somatosensory cortex, usually with incorporation of the hand and arm area to the face area in the case of upper limb amputation; after leg amputation, sensations are referred from genitals to the phantom foot, likewise indicating functional union of those somatotopic areas (Kaas et al., 1983; Sanes et al., 1988; Ramachandran et al., 2007; de Villers-Sidani and Merzenich, 2011).

### Spinal Cord Injuries

Patients with spinal cord injuries often report movement sensation or pain below the level of the spinal lesion, which also amounts to a phantom phenomenon (Melzack, 1990). Siddall and McClelland (1999), in a study of 103 patients, reported movement illusions in nine of them. They found that “four of the nine reported that the legs felt as though they were swinging. The other reports included a sensation of movement in the hands and fingers, including one in which there was a sensation of picking up something between the fingers”. This might be explained by central production of corollary discharges. However, in that same series, patients with phantom limb sensations were not able to voluntarily change phantom limb position.

In spinal cord injuries there are also important plastic changes in the CNS. Topka et al. (1991) demonstrated, by TMS mapping of the motor cortex, that there was enhanced excitability of muscle representation areas of body parts rostral to the spinal cord lesion (e.g., of abdominal muscles).

### Early Stages of Repetitive Strain Injury (RSI)

RSI is commonly found in workers who perform repetitive, low-amplitude movements with little or no visual feedback, such as typing on a computer keyboard. The low movement amplitude decreases the amount of proprioceptive feedback. Thus, there is a discrepancy between intention to move (i.e., corollary discharges), visual and proprioceptive feedback (Harris, 1999).

In a monkey model of RSI, Byl et al. (1997) trained the animals to perform a repetitive task: closing a handpiece against an 8% force (3–400 trials per day, training at 80–90% accuracy). There was a degradation and dedifferentiation of the normally sharply segregated areas of the hand representation in area 3b. Individual fingers did not have separated cortical representation areas anymore, and this interfered with motor control.

Byl et al. (1996) have examined patients with RSI, and have shown defects in kinaesthesia, stereoacuity, and graphaesthesia, suggesting that those patients had changes in cortical representation similar to those in the monkey model.

Central changes and discrepancy between motor intention, visual feedback and proprioception could explain the presence of persistent pain in RSI before any detectable pathological changes in the affected hand.

### Complex Regional Pain Syndrome (CRP)

The first report of possible cortical sensory somatotopic reorganization in patients with CRP was that of McCabe et al. (2003). Five of 16 subjects recruited by them demonstrated referred sensations (RFs) to different body parts during clinical tests of sensation. Such RFs were experienced in real time, were modality specific (touch and pinprick) and were located on the body part immediately adjacent, on Penfield’s cortical homunculus, to the stimulated site. This is similar to RFs described in amputees (Ramachandran et al., 2007) and suggests encroaching of cortical representations of body parts affected by CRP upon adjacent cortical somatotopic areas. One possible explanation for such a phenomenon might be that, due to the greatly hiperexcitable afferences from the limb with CRP, its cortical representation area increases and becomes functionally connected to adjacent areas in the homunculus.

Motor symptoms in CRP include weakness, tremor, dystonia and myoclonia. Maihöfner et al. (2007) demonstrated a significant reorganization of central motor circuits in CRP patients, with an increased activation of primary motor and supplementary motor cortices (SMA) as revealed by functional magnetic resonance imaging, during finger tapping of the affected extremiy. Additionally, the ipsilateral motor cortex showed a markedly increased activation.

## Possible Therapeutic Approaches for Pain Arising from Maladaptive Brain Plasticity

### Restoration of Normal Cortical Somatotopy

Aberrant somatotopy secondary to maladaptive brain plasticity probably results in changes in corollary discharges, leading to sensory-motor incongruities. Depending on the underlying condition, sensory feedback might also be abnormal. Rehabilitation strategies aiming at normalization of cortical somatotopy might also decrease pain. Harris (1999) suggested, for example, that typists suffering from RSI might benefit from the performance of daily exercises involving individual fingers, coupled with sensory stimulation, so as to restore a normal cortical map of the involved hand. Special keyboards allowing for longer finger excursions during typing, as well as keyboard visualization during typing, would also be advisable.

### Mirror Therapy

Mirror therapy has been successfully tried in amputees with chronic phantom pain (Ramachandran et al., 2007) and might also be used in other cases where visual feedback is difficult or impossible (e.g., chronic back pain). According to Ramachandran et al. (2007), in cases of stroke or other conditions leading to movement impairment, the paralysis might be in part learned, and visual feedback of a normally moving limb on a mirror might help the motor system overcome such learned component.

### Neuromodulation Techniques

TMS and tDCS have been tried, with variable success, in cases of chronic pain (Lefaucheur et al., 2001, 2011; Fregni et al., 2006a,b; André-Obadia et al., 2014). The motor cortex has been the most frequent target of all studies, given the success achieved by neurosurgical stimulation (Tsubokawa et al., 1991a,b).

Mechanisms suggested for the beneficial effects of motor cortex stimulation include: (1) stimulation of parallel fibers involved in top-down control of pain perception rather then direct stimulation of motor neurons (Nguyen et al., 2011); (2) indirect stimulation of distant areas, accounting for modulation of emotional aspects of pain (Strafella et al., 2003; Sacco et al., 2014); (3) restoration of defective intracortical inhibition in the motor cortex of chronic pain patients (Lefaucheur et al., 2006); (4) release of endogenous opioids (Maarrawi et al., 2007); and (5) changes in various neurotransmitters in the motor cortex, striatum and limbic system (DosSantos et al., 2016).

On the other hand, the right dorsolateral prefrontal cortex (DLPC) has been mainly targeted, with inhibitory stimulation (either low-frequency repetitive transcranial magnetic stimulation (rTMS) or cathodal tDCS) to treat depression. One study applied high-frequency rTMS to the left DLPC and found a beneficial effect on capsaicin-induced pain (Sacco et al., 2014). However, given the neuroradiological evidence of a role of the right DLPC in the continuous monitoring of sensory-motor incongruities (Fink et al., 1999), that same strategy might also decrease chronic pain. Another interesting possibility for neuromodulation would be to enhance the neuroplastic effects of exercises aimed at restoring normal brain maps by concomitant tDCS, as has already been done in other forms of motor learning (Hashemirad et al., 2015).

## Conclusion

In conclusion, motor cortex stimulation for treatment of chronic pain with non-invasive neuromodulatory techniques such as rTMS and tDCS had variable degrees of success. A better understanding of the effects of increasing M1 excitability by these techniques upon the physiology of the complex pain neuromatrix is clearly needed.

Modulation of motor corollary discharges might be one such mechanism, and there is evidence that neuromodulatory techniques may have diferent effects on the populations of neurons that generate motor output in M1 and on those neural structures that are involved in generating corollary discharges (Voss et al., 2007). If there is indeed a link between modulation of corollary discharges and analgesia, concomitant stimulation of the dorsal prefrontal area might increase the beneficial effect, since this area has been shown to produce more motor corollary discharges than M1 after rTMS (Christensen et al., 2010). Moreover, left DLPC stimulation by rTMS has been shown to have an analgesic effect of its own, even in the absence of simultaneous M1 stimulation (Sacco et al., 2014).

However, new neuromodulatory strategies might be attempted. More specifically, since PET studies have implicated the right DLPC as a monitoring center for sensory-motor mismatch, it would be interesting to investigate a possible beneficial effect of inhibiting this area, using either low-frequency rTMS or cathodal tDCS, in cases of chronic pain. In fact, Graff-Guerrero et al. (2005) described an analgesic effect of 1 Hz rTMS of the right DLPC. It might have been the result of direct inhibition of this area or of reciprocal inhibitory connections between right and lef DLPC through the corpus callosum. Further neuromodulation studies targeting the right DLPC might eventually help to clarify this issue.

Finally, when the underlying disease causes potentially reversible changes of cortical somatotopic maps, as in RSI cases, exercise programs to restore normal cortical representation of the involved body parts might benefit from adjuvant neuromodulatory treatments, such as tDCS.

## Author Contributions

JPB-N wrote this article. The author confirms being the sole contributor of this work and approved it for publication.

## Conflict of Interest Statement

The author declares that the research was conducted in the absence of any commercial or financial relationships that could be construed as a potential conflict of interest.
